# Comparison of bench test results measuring the accuracy of peak flow meters

**DOI:** 10.1186/s12890-019-0837-3

**Published:** 2019-04-08

**Authors:** Cristiano VanZeller, Andrew Williams, Ian Pollock

**Affiliations:** 1 Royal London Hospital, London, UK; 2 Guy’s Hospital, London, UK; 3 St Mary’s Hospital, London, UK

**Keywords:** Peak flow meter, Accuracy, CE, Asthma

## Abstract

**Background:**

The study evaluates and compares the accuracy of nine peak flow meters (“PFMs”) and spirometers that are currently available in Europe and have Conformité Européene (“CE”) marking. The CE marking is a manufacturer’s declaration that their product complies with European health regulations and it is a requirement for marketing medical devices in Europe.

**Methods:**

The nine devices were selected as they all had received or were in the process of receiving CE approval in Europe and were readily obtainable. The devices were bench tested following the ISO 23747:2015 accuracy guidelines for medical devices measuring peak flow. All standards, including accuracy, from these guidelines must be met to obtain CE marking.

This study was performed with a certified piston pump testing apparatus. The apparatus chosen was the pulmonary waveform generator manufactured by Piston Medical Ltd. Using predefined flow (time) and volume (time) waveforms, peak flow meters and spirometers were tested for validation and calibration. Three CE guideline tests were utilised, and standards require that all three tests are passed for the device to obtain certification.

**Results:**

Of the nine devices that were tested, two passed and seven failed. The devices that passed the tests were the Smart Peak Flow® and the Mini Wright®.

**Conclusions:**

A high percentage of devices failed accuracy testing in this study. This is a concern as the CE marking is a manufacturer’s certification documenting the accuracy, reliability and safety of devices. Of the seven devices that failed all have the CE marking. All tested devices are on the market in Europe based upon studies conducted by each of the manufacturers. The data used to obtain CE certification of these devices, however, are not in the public domain.

## Background

Peak flow meters are invaluable tools in diagnosing and managing respiratory conditions especially asthma.

The measurement of peak expiratory flow rate (“PEFR”) using a peak flow meter (“PFM”) was established by a British bioengineer, Martin Wright, who invented the first PFM in 1956. Since then, major international collaborations and disease-specific guidelines, both in the United Kingdom [1] and internationally [2], have incorporated PEFR measurement into their recommendations.

Many different mechanical peak flow meters, which all utilise the same basic mechanics but otherwise vary greatly in design, have been developed since Martin Wright’s invention and are available on the market. Digital peak flow meters have more recently become available and permit replacing written records of results with direct electronic storage and data sharing capability.

CE stands for the French phrase “Conformité Européene” which means “European Conformity”. In Europe, since 1985, CE marking has been used to signify that these products comply with the essential requirements of the relevant European health, safety and environmental protection legislation. Certification is gained by following guidelines through either a self-certification or an independent process depending on the risk of the product to individuals. Products that are certified typically display the CE mark.

This bench test study used the specific accuracy related sections of the applicable guideline in order to evaluate and compare the accuracy of various PFMs and spirometers currently distributed in the European market and which hold a CE marking*.*

## Methods

This bench test study was carried out on June 28th 2017 in Budapest, Hungary. Nine PFMs or spirometers with CE marking either existing or pending were selected. These devices are the only CE marked devices available in Europe. The PFMs and spirometers were then tested in one day under identical laboratory conditions specifically evaluating only PEFR.

In order to determine the accuracy of the devices tested in this study, the applicable CE guidelines of ISO 23747:2015 as developed by The International Organization for Standardization (“ISO”) were followed. This ISO standard covers all medical devices that measure peak expiratory flow rate in spontaneously breathing humans either as part of an integrated lung function medical device or as a stand-alone medical device. The existence of this standard is important for clinicians to diagnose and monitor lung and airway conditions by ensuring that all medical devices used for such purposes meet minimum levels of safety and performance. It is important that for each device all conditions described in this standard are met in order to attain CE conformity and approval.

**Fig. 1:**
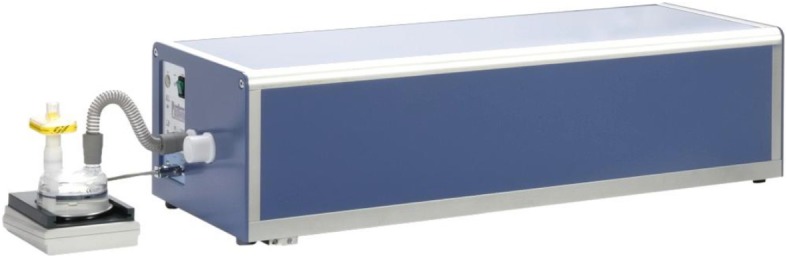
The pulmonary wave generator PWG-33 manufactured by Piston Medical Limited

The devices were tested using a pulmonary waveform generator (“PWG”). A PWG is an apparatus that generates a waveform of known peak flow rate that is discharged through each peak flow meter. The measured output is compared with the set reference peak flow rate in order to determine the accuracy. PWGs are used for developing and testing spirometers and other flow/volume measuring devices. The requirements for the testing apparatus as the known source of airflow are described in detail in ISO 23747:2015. For this study, a CE certified piston pump testing apparatus shown in Fig. 1, the PWG-33 manufactured by Piston Medical Limited, was used.

The PWG-33 provides predefined flow (time) and volume (time) waveforms for the validation and calibration of PFMs, spirometers and other equipment measuring flow and volume. To carry out the measuring procedure, the tested device was connected to the airflow outlet of the PWG apparatus using a rigid smoothbore coupling shorter than 100 mm in length and the device was set up according to the instructions for use.

The following information was recorded for each device in assessment report forms:

- Date and time of the tests
- Details about the device and the air flow source
- Ambient conditions
- Details about the testing software
- Description of the tests
- Visual documentation of the testing process
- The report generated by the PWG containing the recorded flow rates

ISO 23747:2015 defines a number of test profiles based upon the intended patient population based upon published data. The performance looks at three characteristics:

- Error, linearity and repeatability
- Frequency response
- Resistance

Three basic tests were performed on each device to determine their accuracy:

- T1: Determination of error, repeatability and resistance to peak expiratory output at flow rates of 100, 150, 200, 300, 450, 600 and 720 l/min.
- T2: Determination of error at body temperature, ambient pressure, saturated (with water vapor) conditions (body temperature (37 °C), at the measured pressure when saturated with water vapour) at flow rates of 300 and 600 l/min.
- T3: Determination of frequency response at flow rates of 180, 360 and 540 l/min.

The pass/fail criteria for tests T1, T2 and T3 are clearly defined in ISO 23747:2015. Any deviation of the peak flow meter reading which is less than the sum of the stated permissible errors and the known error of the test apparatus is considered as a pass. Any deviation over the permissible errors is considered as a fail.

## Results

Air Smart Spirometer®

- Pond Healthcare Innovation AB
- Spirometer digital CE 0598 LOT: B010
- T1: PASS T2: PASS T3: FAIL
- OVERALL RESULT - FAIL

AirZone®

- Clement Clarke International Ltd.
- Peak flow meter mechanical CE 0120 LOT: M4200020K
- T1: FAIL T2: PASS T3: FAIL
- OVERALL RESULT - FAIL

eMini Wright®

- Clement Clarke International Ltd.
- Peak flow meter digital CE 0120 SERIAL: SN00500
- T1: PASS T2: PASS T3: FAIL
- OVERALL RESULT - FAIL

Medi®

- Medicareplus International Ltd.
- Peak flow meter mechanical CE 0598 LOT: 1342
- T1: FAIL T2: FAIL T3: FAIL
- OVERALL RESULT - FAIL

Mini Wright®

- Clement Clarke International Ltd.
- Peak flow meter mechanical CE 0120 LOT: M004151K
- T1: PASS T2: PASS T3: PASS
- OVERALL RESULT - PASS

MIR Smart One®

- MIR Medical International Research Srl.
- Spirometer digital CE 0476 SERIAL: A23-A000485
- T1: PASS T2: PASS T3: FAIL
- OVERALL RESULT - FAIL

Philips PersonalBest®

- Respironics Respiratory Drug Delivery Ltd
- Peak flow meter mechanical LOT: 16A005
- T1: PASS T2: PASS T3: FAIL
- OVERALL RESULT – FAIL

Smart Peak Flow®

- Smart Respiratory Products Ltd.
- Peak flow meter digital - prototype
- T1: PASS T2: PASS T3: PASS
- OVERALL RESULT - PASS

Vitalograph®

- Vitalograph (Ireland) Ltd.
- Peak flow meter mechanical CE 0086 LOT: 1408
- T1: FAIL T2: FAIL T3: FAIL
- OVERALL RESULT - FAIL

## Discussion

As is the case with all medical devices, accuracy is a critical characteristic of a PFM.

PFMs are in widespread use, both in general practice and in hospitals by various health professionals as a tool to help characterise and diagnose respiratory problems. Clinicians are advised to develop self-management plans with patients that incorporate actions using peak flow measurements in addition to assessing their symptoms. Many asthma guidelines, including those of the British Thoracic Society [1], recommend that patients, following their personalised Asthma Action Plan, use peak flow meters on a regular basis to monitor their condition. The Global Initiative for Asthma (GINA) [2] states that “guided self-management education” is “highly effective in improving asthma outcomes” and that there are three essential components to self management which are self-monitoring, written action plans and regular medical review. When comparing these factors, written action plans based on PEFR monitoring and symptoms-based monitoring are equivalent in reducing hospitalisation, emergency room and unscheduled doctor visits as well as nocturnal asthma symptoms in adults [3], whilst in children there is evidence that a symptom-focussed approach is slightly superior [4]. There is no conclusive evidence that smartphone or tablet apps support the use of self-management plans [5].

PFM accuracy is also important as these devices are utilised in asthma clinical studies. The use of electronic diaries for PEFR monitoring, such as those in electronic PFMs, is now routine in clinical research studies [6].

Analyses of asthma deaths note various avoidable contributing factors, including the lack of self-management plans incorporating the monitoring of peak flows [7]. Clinically significant changes in patient recorded peak flow rates influence changes in treatments and may result in patients seeking emergency medical care. Many patients have poor compliance with measurement of PEFR when clinically well and not everyone remembers their best PEFR, making assessment of the severity of acute exacerbations difficult. Accurate measurement of PEFR allows calculation of their predicted values and can further support therapeutic decisions in certain circumstances.

Accurate PFMs may thus contribute to reducing both under and overtreatment, to improving diagnosis, to improving clinical outcomes [1], and also the accuracy of clinical trials, in which PEFRs are frequently endpoints for treatment efficacy [6].

Seven of the nine devices in this study failed at least one of the three tests. As a result, in this study, these seven devices failed under the ISO 23747:2015 guidelines. Only one of the mechanical peak flow meters, the Mini Wright®**,** passed with the other four mechanical devices failing. Only one of the digital peak flow meters, the Smart Peak Flow®, passed with the other three digital devices failing. These would be the only two devices that would comply with the minimum performance requirements to achieve the CE marking and can thus provide accurate results that clinicians and patients can confidently rely on. All of the devices that failed are currently being marketed in Europe. It is of concern that there are a considerable number of apparently inaccurate devices available on the European market, with a possible impact on patient care. Patients and differing healthcare organisations may use different devices to measure PEFRs and the heterogeneity in the accuracy of marketed devices may adversely impact on the clinical management of exacerbations.

One of the strengths of this study was that it had a clear methodology that complied with the accuracy standards recommended for achieving the CE mark. There is otherwise no publicly available data on many of the PFMs that were tested.

Previous published studies including those by Nazir et al. [8] and by Takara et al. [9]have shown that there is variability in accuracy between PFMs when tested using human subjects.

Nazir et al. [8] found significant differences when double blinded testing three peak flow meters in 409 human subjects. Variability in accuracy with the same device was also demonstrated in this study.

Takara et al. [9] studied five PFMs in 68 human subjects and reported that one of the PFMs underestimated peak flows while another device overestimated peak flows. The authors of these papers have expressed clinical concern with the results seen in their studies.

There are examples of studies using pulmonary wave form generators to test spirometers that have demonstrated potential clinically important consequences of their under-performance. A published study by Hegewald et al. [10] used bench testing to show that only 1 of 17 spirometers used in primary care in the United States met the ATS definition of accuracy and precision when tested using a pulmonary wave form generator. Whilst seven (7/17) of the devices had mean errors exceeding the ATS recommended repeatability-criteria between maneuover, when the mean error across all devices was applied to a set of obstructed patients, 28% were reclassified as non-obstructed.

## Conclusions

We found that many peak flow meters and spirometers that are available in Europe under the CE marking certification failed testing using the accuracy guidelines of ISO 23747:2015. Of the nine devices tested only two, the MiniWright® and the Smart Peak Flow®, passed and the other seven devices failed.

This result is a concern as the CE marking is a manufacturer’s certification proving accuracy of devices. The devices that failed in this study have the CE marking and are on the market in Europe.

## Abbreviations

CE: Conformité Européene
ISO: The International Organization for Standardization
PEFR: Peak expiratory flow rate
PFM: Peak flow meters
PWG: Pulmonary waveform generator
